# Efficacy of ultrasound-guided forearm nerve block versus forearm intravenous regional anaesthesia in patients undergoing carpal tunnel release: A randomized controlled trial

**DOI:** 10.1371/journal.pone.0246863

**Published:** 2021-02-19

**Authors:** Hassanin Jalil, Florence Polfliet, Kristof Nijs, Liesbeth Bruckers, Gerrit De Wachter, Ina Callebaut, Lene Salimans, Marc Van de Velde, Björn Stessel

**Affiliations:** 1 Department of Anaesthesiology and Pain Medicine, Jessa Hospital, Hasselt, Belgium; 2 I-BioStat, Data Science Institute, Hasselt University, Hasselt, Belgium; 3 Department of Orthopedic Surgery, Jessa Hospital, Hasselt, Belgium; 4 Faculty of Medicine and Life Sciences, UHasselt, Agoralaan, Diepenbeek, Belgium; 5 Department of Cardiovascular Sciences, KULeuven, Leuven, Belgium; 6 Department of Anaesthesiology, University Hospital, Leuven, Belgium; Cleveland Clinic, UNITED STATES

## Abstract

**Background and objectives:**

Distal upper extremity surgery is commonly performed under regional anaesthesia, including intravenous regional anaesthesia (IVRA) and ultrasound-guided forearm nerve block. This study aimed to investigate if ultrasound-guided forearm nerve block is superior to forearm IVRA in producing a surgical block in patients undergoing carpal tunnel release.

**Methods:**

In this observer-blinded, randomized controlled superiority trial, 100 patients undergoing carpal tunnel release were randomized to receive ultrasound-guided forearm nerve block (n = 50) or forearm IVRA (n = 50). The primary outcome was anaesthetic efficacy evaluated by classifying the blocks as complete vs incomplete. Complete anaesthesia was defined as total sensory block, incomplete anaesthesia as mild pain requiring more analgesics or need of general anaesthesia. Pain intensity on a numeric rating scale (0–10) was recorded. Surgeon satisfaction with hemostasis, surgical time, and OR stay time were recorded. Patient satisfaction with the quality of the block was assessed at POD 1.

**Results:**

In total, 43 (86%) of the forearm nerve blocks were evaluated as complete, compared to 33 (66%) of the forearm IVRA (p = 0.019). After the forearm nerve block, pain intensity was lower at discharge (-1.76 points lower, 95% CI (-2.92, -0.59), p = 0.0006) compared to patients treated with forearm IVRA. No differences in pain experienced at the start of the surgery, during surgery, and at POD1, nor in surgical time or total OR stay were observed between groups. Surgeon (p = 0.0016) and patient satisfaction (p = 0.0023) were slightly higher after forearm nerve block.

**Conclusion:**

An ultrasound-guided forearm nerve block is superior compared to forearm IVRA in providing a surgical block in patients undergoing carpal tunnel release.

**Trial registration:**

This trial was registered as NCT03411551.

## Introduction

Regional anaesthesia is regularly performed in upper extremity surgery. With the ultrasound, nerve blocks are now more reliable and safe to perform. As a consequence, ultrasound-guided proximal brachial plexus blockade is considered the gold standard in regional anaesthesia during distal upper extremity surgery [1]. Distal peripheral nerve blocks provide extra advantages such as the avoidance of complications like pneumothorax and phrenic paralysis. Second, these distal peripheral blocks can preserve the motor function of the digits as well as the motor function of the more proximal muscles [2, 3]. It is suggested that the preservation of motor function after distal nerve block is associated with a higher satisfaction rate compared to proximal brachial plexus block [4]. It may even protect the patient from injury [5]. Additionally, compared to general anaesthesia or intravenous regional anaesthesia (IVRA), the use of a block room to perform peripheral nerve block techniques (outside of the operating room) may improve operating theatre efficiency [6]. On the downside, it is not possible to prevent tourniquet pain (when needed for surgery) with distal (from the elbow) nerve blocks since the cutaneous innervation of the proximal part of the forearm and the upper arm is not blocked. However, the tourniquet seems to be better tolerated in surgeries with short timespans [7]. Furthermore, patients can feel discomfort due to the multiple injections needed to block the nerves in a distal nerve block.

In our hospital, single-cuff forearm IVRA is the most frequently used anaesthetic technique in distal upper extremity surgery. Forearm IVRA is as efficient in providing a surgical block as compared to a conventional upper arm IVRA, even with a lower, non-toxic dose of local anaesthetic [8]. Therefore, it has a better safety profile than conventional IVRA. However, due to the mechanism of action, the density of the surgical block produced by IVRA may be questioned and it doesn´t offer postoperative analgesia [9].

This randomized controlled superiority trial was set out to compare ultrasound-guided forearm median and ulnar nerve block and forearm IVRA in patients undergoing carpal tunnel release. We hypothesized that a forearm nerve block would be superior to forearm IVRA in terms of analgesic efficacy, i.e. producing a surgical block.

## Material and methods

### Trial design

In this prospective, mono-center, observer-blinded, randomized controlled superiority trial, the efficacy of an ultrasound-guided peripheral nerve block versus a forearm IVRA was assessed in 100 patients undergoing carpal tunnel release between January 2018 and July 2019. This study was approved by the ethical committee of the Jessa Hospital, Hasselt, Belgium (Chairperson Dr. K. Magerman, registration number 17.118/anesth17.03, B243201734692) on 22^nd^ December 2017, registered on ClinicalTrials.gov (NCT03411551) and executed according to the Declaration of Helsinki. This study is reported according to the Consolidated Standards of Reporting Trials (CONSORT) statement [10].

### Eligibility criteria

Adult patients undergoing carpal tunnel release with an ‘American Society of Anaesthesiologists’ (ASA) physical status of 1 to 3 were eligible. Exclusion criteria included an ASA status >3, <18 years old, bilateral surgery, a BMI ≥40, a local site infection, a history of neurological disorders, chronic pain symptoms, diabetes mellitus, allergy to local anaesthetic, a coagulopathy, prior ipsilateral arm surgery and the inability to understand and adhere to the study design. Written informed consent was obtained from all patients before inclusion in the study.

### Randomization

Participants were randomly assigned in a 1:1 ratio to an ultrasound-guided forearm nerve block (n = 50) or a forearm IVRA (n = 50). Block randomization with a block size of 6 was performed using a computer-generated random allocation sequence [11], created by the study statistician. Allocation numbers were sealed in opaque envelopes, which were opened in sequence by an independent anaesthesiologist who was not involved in the assessment of outcomes.

### Interventions and study procedures

All patients received intravenous access in the contralateral arm, supplementary oxygen, and standard monitoring (non-invasive blood pressure, electrocardiogram, and saturation measurements) in the regional anaesthesia block room. Furthermore, they received preoperatively intravenous paracetamol 15mg/kg (max 1gram), ketorolac 0.5mg/kg (max 30mg) and dexamethasone 0.1mg/kg (max 5mg). Patients allocated to the forearm IVRA group also received a 22-gauge indwelling intravenous catheter in a dorsal vein of the ipsilateral hand. This vein was catheterized as distally as possible to ensure the optimal distribution of the lidocaine. A band-aid was placed on the dorsal side of the ipsilateral hand of patients in the peripheral nerve block group to achieve the blinding of observers. All peripheral nerve blocks were performed in the regional anaesthesia block room. Forearm IVRA was performed in the operation theatre.

#### Ultrasound-guided peripheral nerve block group

**Fig 1:**
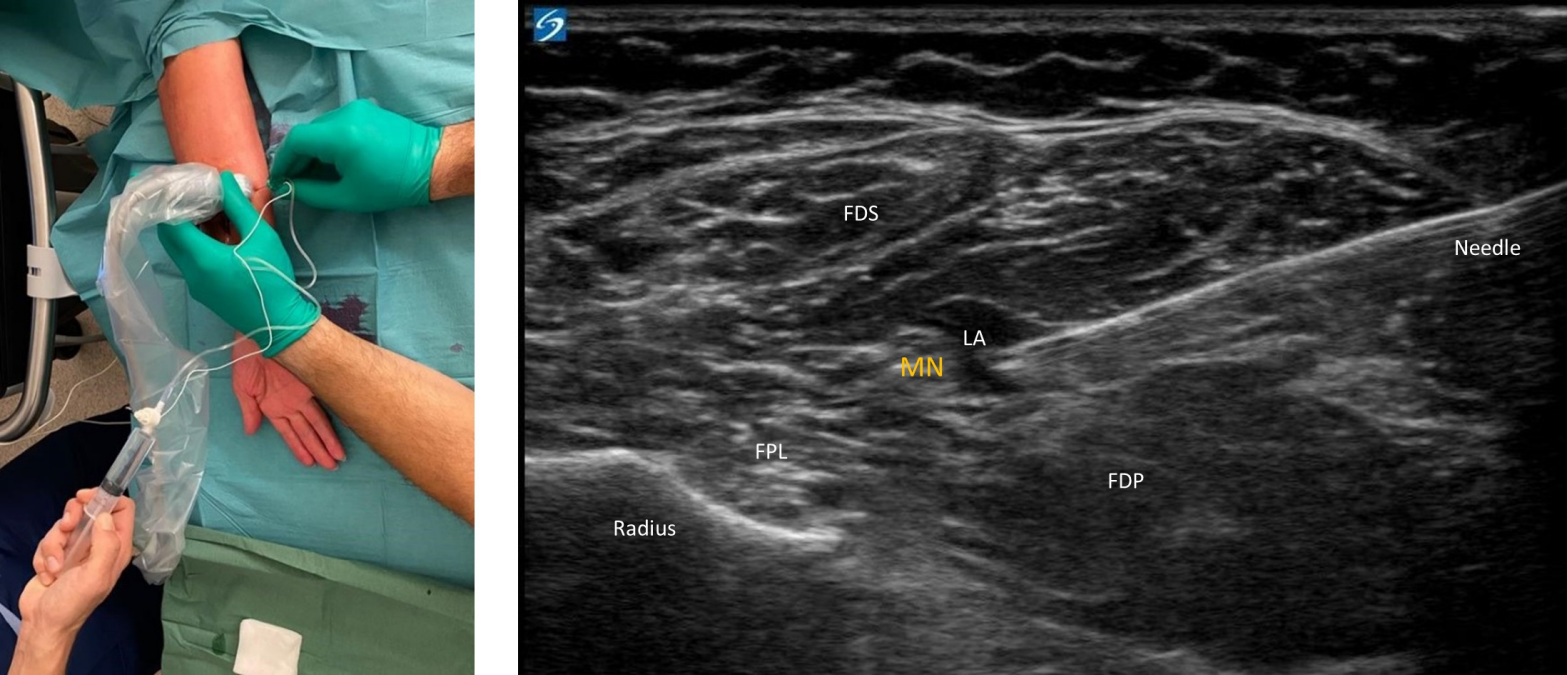
Localization of the median nerve. Abbreviation: *MN*, *median nerve; FPL*, *flexor pollicis longus; FDS*, *flexor digitorum superficialis; FDP*, *flexor digitorum profundus*.

Patients randomized to an ultrasound-guided peripheral nerve block were placed in the supine position in the regional anaesthesia block room. The ipsilateral arm was abducted and rotated externally. A Sono Site Xporte ultrasound machine with a high-frequency linear transducer HFL38 (13-6MHz) and a 22G Stimuplex Ultra 50 mm (B. Braun Medical Inc, Melsungen, Germany) needle were used for all blocks. To localize the median nerve, the ultrasound transducer was placed in a transverse orientation proximally to the wrist cease at mid-forearm level, with a slight tilt distally towards the hand (Fig 1). The median nerve appears as an oval hyperechoic structure in a fascial plane in between the deep and superficial flexor muscles of the hand. An in-plane ultrasound-guided medial-to-lateral puncture was made and 3ml of 2% lidocaine was injected within the fascia that envelopes the median nerve.

**Fig 2:**
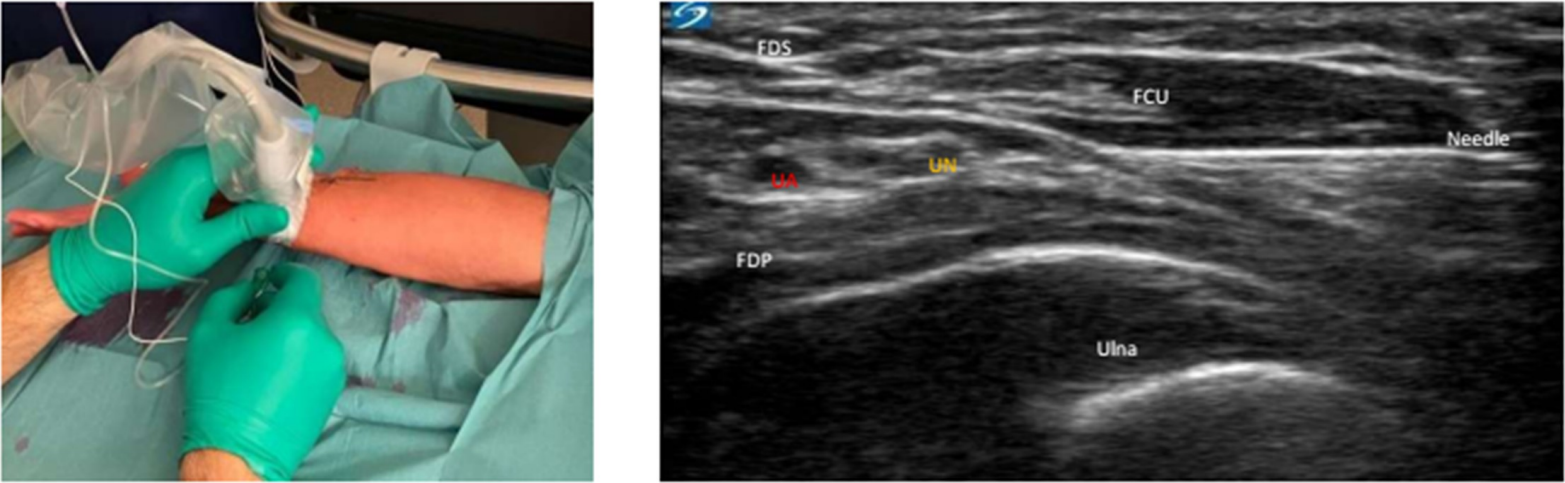
Localization of the ulnar nerve. Abbreviation: *UN*, *ulnar nerve; UA*, *ulnar artery; FCU*, *flexor carpi ulnaris; FDS*, *flexor digitorum superficialis; FDP*, *flexor digitorum profundus*.

To perform the ulnar block, an ultrasound transducer was placed in a transverse orientation over the ulnar side of the distal forearm (Fig 2). After identification of the ulnar artery, the ulnar nerve is imaged as a triangular or oval. Depending on the available space, an in-plane ultrasound-guided medial-to-lateral or proximal-to distal puncture was made and 3 ml of 2% lidocaine was infiltrated into the fascial plane to block the ulnar nerve. Finally, we applied a circumferential subcutaneous infiltration of 4 ml 2% lidocaine with a 25G needle at the radial side of the wrist, 2cm proximal to the styloid process of the radius to block the terminal sensory branches of the radial, musculocutaneous, and medial antebrachial cutaneous nerves that may reach the palmar crease [12].

#### Forearm IVRA group

Patients randomized to a forearm IVRA were placed in the supine position in the operation theatre. After placement of the forearm tourniquet and exsanguination of the limb distal to the cuff by applying an Esmarch’s bandage starting from the fingertips, the cuff is inflated to a pressure of 300 mmHg. Subsequently, tourniquet failure is ruled out by observing the distal circulation and 25ml 0.5% lidocaine [13] is slowly injected through the intravenous cannula on the dorsum of the hand.

#### Post-operative pain therapy

Post-operative pain medication in the hospital included paracetamol 15mg/kg (max 1gr) 1x/6h and ketorolac 0.5mg/kg (max 30mg) 1x/8h, while tramadol served as rescue medication for all patients. Post-operative pain medication at home included per oral paracetamol 15mg/kg (max 1gr) 1x/6h and ibuprofen 600mg 1x/8h whenever necessary.

### Outcomes

The primary outcome of this study was the anaesthetic efficacy of both techniques, which was evaluated by the blinded surgeon before the surgery using a forceps to assess the quality of the block on the ulnar nerve and median nerve. Complete anaesthesia was defined as a full (grade 1) or partial motor block (grade 2) and no pain (i.e. a total sensory block with pinprick). Incomplete anaesthesia was defined as a partial motor block and mild pain requiring more intravenous analgesics (grade 3) or an incomplete motor block with severe pain requiring general anaesthesia (grade 4). In the case of grade 3, alfentanil 5 mcg/kg IV and/or sufentanil 0.05–0.1 mcg/kg IV were administered and the surgeon was asked to locally inject an additional dose of 4 ml 2% lidocaine at the site of the surgery [14]. In the case of grade 4, conversion to general anaesthesia was performed. The quality of the block was tested by the blinded surgeon before surgery. The sensory block was assessed by pinching the skin with a forceps in the ulnar nerve and median nerve cutaneous territory. Motor block was assessed by thumb opposition (median nerve) and thumb adduction (ulnar nerve). Secondary outcome measures included procedural pain during the performance of the block and injection of the local anaesthetic, pain at incision, and every 10 minutes during surgery (including tourniquet pain), pain at discharge, and pain at POD1. The pain was assessed with an 11-point Numerical Rating Scale (NRS), with 0 indicating no pain and 10 indicating the worst possible pain. Furthermore, surgical time and total operation room (OR) stay time were recorded. Surgical time was defined as the time from incision to surgical completion and application of dressings. Total OR stay time was defined as departure time from the OR minus arrival time in the OR. Finally, satisfaction with the quality of the surgical field, based on the degree of incomplete hemostasis was assessed by the surgeon at the end of surgery with a 7-point Likert Scale, with 1 indicating extremely dissatisfied and 7 indicating extremely satisfied and patient satisfaction with perioperative analgesia was evaluated by telephone call at POD 1 with a 7-point Likert Scale. The 7-point Likert scale can be found in the study protocol (S1 and S2 Files).

### Sample size

The study was powered for the primary outcome, i.e. the quality of the block to demonstrate the superiority of a forearm nerve block compared to forearm IVRA. The quality of the block was redefined in a complete block (grade 1 or 2) and incomplete block (grade 3 or 4) creating a binary primary outcome. For the sample size calculation, a clinically relevant difference of 20% between the two groups was used. Based on a retrospective analysis of unpublished data from our hospital, we expected 75% of patients having a complete block (grade 1 or 2) after a forearm IVRA. Based on a previous study [7], we also expected 95% of patients having a complete block (grade 1 or 2) after an ultrasound-guided peripheral nerve block. A sample size of 47 patients per group achieves 80% power to detect a difference of 20% using a two-sided Chi-Square Test with a significance level (alpha) of 0.05. To account for a possible 5% drop-out rate, the sample size was increased to 50 patients per group.

### Blinding

Outcome-assessors (surgeon and study assistant) were blinded to treatment allocation (observer-blinded study). To achieve observer blinding, surgeons and study assistants were not allowed in the regional anaesthesia block room and were asked to leave the operation theatre at the end of each procedure. They were only allowed to enter the operation theatre after full preparation of the next patient. Full preparation included placement of a single-cuff tourniquet on the forearm in both groups, block placement, and subsequent removal of the 22-gauge intravenous catheter with the placement of a band-aid in the forearm IVRA group, preparation of the skin with an antiseptic solution, and finally placement of surgical drapes. These drapes also covered all nerve block sites, only uncovering surgical sites, i.e. wrist and hand, to ensure observer blinding. The single tourniquet, 5cm in width, was placed 5 cm distal to the medial epicondyle of the humerus. A band-aid was already placed on the dorsal side of the ipsilateral hand of patients enrolled in the peripheral nerve block group at the regional anaesthesia block room.

### Statistical analysis

All primary and secondary endpoints were analyzed by an independent statistician on an intention to treat (ITT) basis according to the guidelines for superiority studies. Descriptive statistics were presented as frequencies and percentages of the total number of patients for categorical variables, while numerical variables were presented as medians and interquartile ranges. Group comparison for the binary primary endpoint was performed using a Chi-square test. Mann-Witney U tests were used to compare the groups in terms of surgical time, total OR stay time, the satisfaction of the surgeon, and the satisfaction of the patient. For the repeated NRS pain scores, a linear mixed-effect model was used to assess the significance of time (at the start of the surgery, during surgery, at discharge, and on POD1), group (forearm IVRA, peripheral nerve block), and their interaction on variations in NRS. A random patient effect was used to account for correlations across multiple within-patients NRS outcomes. For most patients (87 out of 100) the surgery took less than 10 minutes and as a result, their pain score during surgery is not available. The linear mixed model can handle incomplete data and valid inferences are obtained under the assumption of missingness at random. QQ-plots for the studentized raw and conditional residuals reveals mild deviations from normality. Although linear mixed models provide reliable estimates under departures from the normality assumption we also implemented a mixed proportional odds model for ordinal data to confirm the conclusions of the linear mixed model. Based on the models for repeated data, pairwise group comparisons were made at each time point, with a Bonferroni correction to keep the type I error at 5% overall.

Besides one missing patient satisfaction score at POD1, there were no missing observations in the primary and secondary endpoints (except the NRS score as indicated above). All hypotheses were tested as two-sided hypotheses at a significance level of 5%. All statistical analyses were performed with SPSS 25.0 (IBM® SPSS® Inc, Chicago, Illinois, USA). All graphs were made using Prism 7.0 (Prism®, GraphPad Software, Inc, La Jolla, California, USA).

## Results

**Fig 3:**
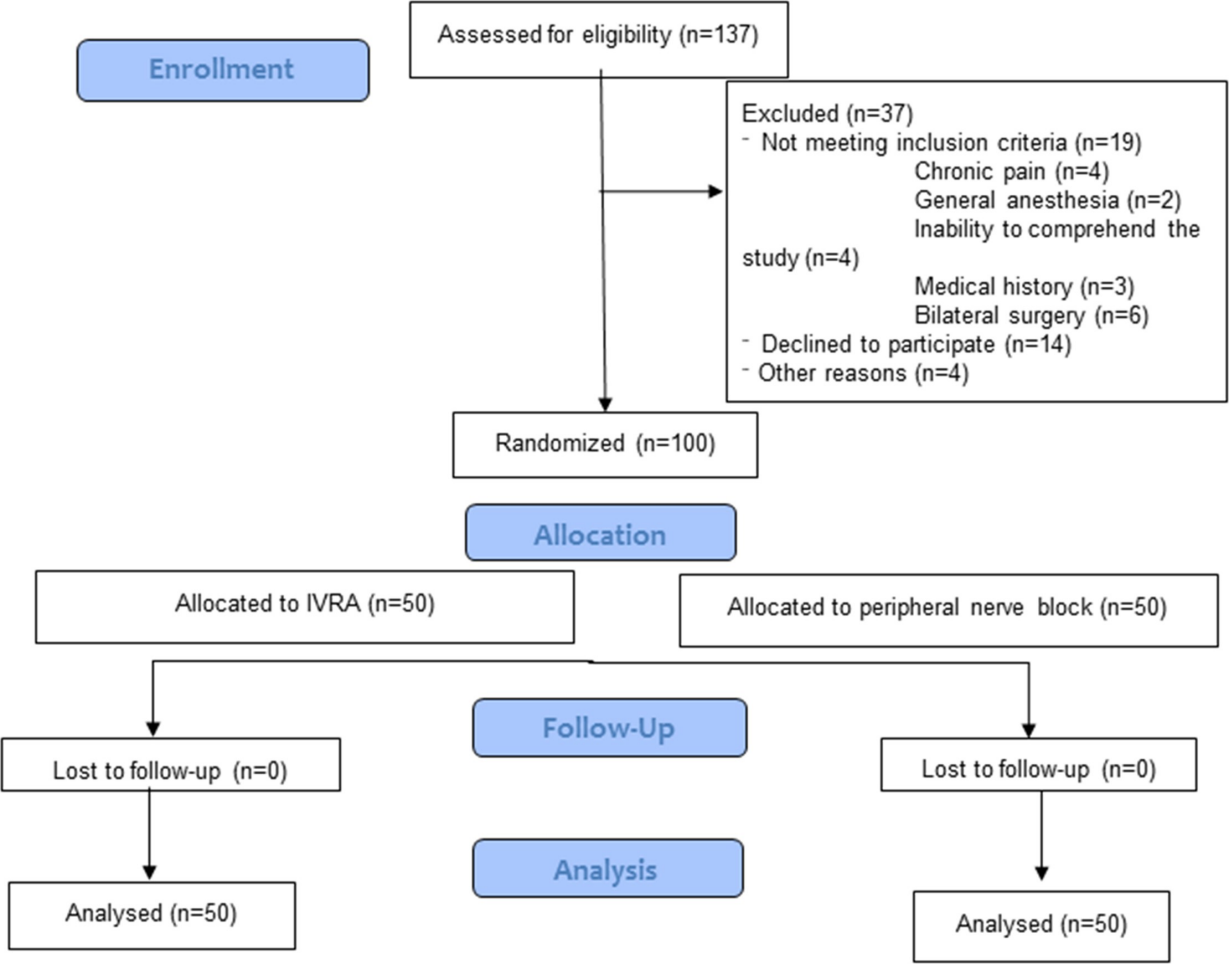
Consort flow chart. *Abbreviation*: *IVRA*, *intravenous regional anaesthesia*.

A CONSORT flow chart of patient selection and exclusion is shown in Fig 3. In total, 137 patients were screened for eligibility between 17^th^ of January 2018 and 10^th^ July 2019, of which 37 patients were excluded due to refusal to participate (n = 14), not meeting the inclusion criteria (n = 19), or other reasons (n = 4).

Patient characteristics are presented in Table 1. In the Forearm IVRA group, 54% of patients were male versus 36% in the Peripheral nerve block.

**Table 1:** Patient characteristics.

	Forearm IVRA	Peripheral nerve block
Gender (male/female)	27/23 (54%)	18/32 (36%)
Age (years)	55.50 (47.00–69.00)	57.50 (49.00–72.00)
BMI	28.39 (24.97, 30.06)	27.30 (23.81, 31.39)
ASA	1 (1, 2)	1 (1, 2)
Side of surgery (left/right)	21/29 (42%)	25/25 (50%)

Data are expressed as numbers (%). Age is expressed as median and interquartile range.

The data for the primary endpoint, i.e. quality of the block as well as the data of the secondary endpoints are presented in Table 2. Ultrasound-guided forearm nerve block was associated with a greater chance of a complete block compared to forearm IVRA (86% vs 66%; p = 0.019; Odds Ratio = 3.16 (95% CI [1.18; 8.52]), Relative Risk = 1.30 (95% CI [1.04; 1.64]) and Risk Difference = 20% (95% CI [4; 36]). Conversion to general anaesthesia (grade 4) was not observed in either treatment group.

**Table 2:** Primary and secondary endpoints.

Variable	Forearm IVRA	Peripheral nerve block	p-value
Quality of the block			
Complete block (grade 1 and 2)	33 (66%)	43 (86%)	**0.019**
Incomplete block (grade 3 and 4)	17 (34%)	7 (14%)	
Surgical time (minutes)	6 (5–8)	6 (5–9)	0.91
Total OR time (minutes)	24 (20–29)	24 (20–29.25)	0.95
Satisfaction of the surgeon	5 (4–6)	6 (6–7)	**0.0016**
Satisfaction of the patient	6 (5–7)	6 (6–7)	**0.0023**

Data are expressed as numbers (%) or as median (IQR).

There was no difference in surgical time nor in total OR stay time between both groups (Table 2).

The surgeon was slightly more satisfied with the visibility of the surgical field when patients after ultrasound-guided peripheral nerve block compared to a forearm IVRA. Patients in the ultrasound-guided peripheral nerve block were slightly more satisfied with perioperative analgesia compared to patients randomized to a forearm IVRA (Table 2). At POD 1, none of the patients reported an adverse event related to anaesthesia (including neurologic deficit).

**Fig 4:**
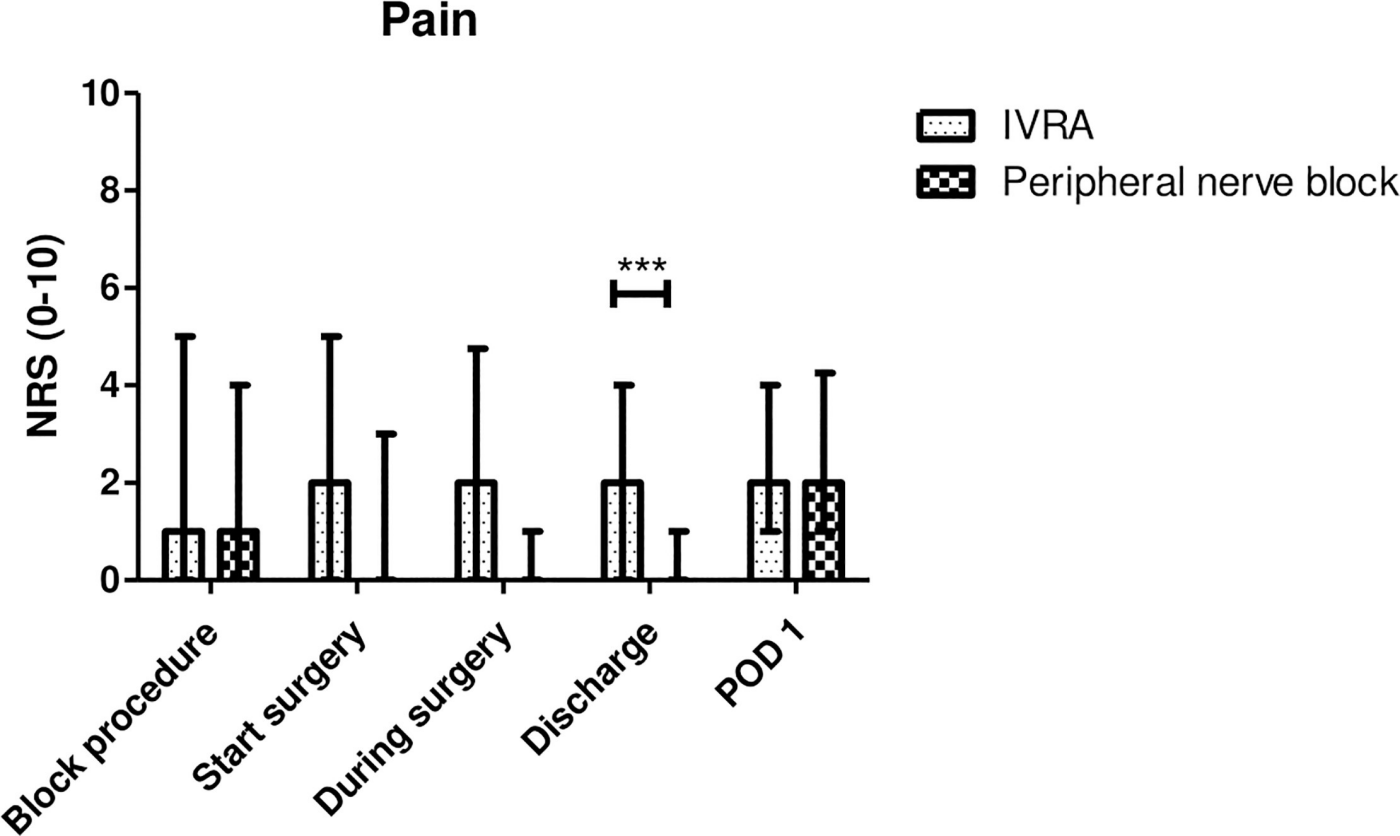
Pain Intensity reported by patients on a Numerical Rating Scale (NRS) at different time points (median and interquartile range). *** p<0.001. *Abbreviation*: *IVRA*, *intravenous regional anaesthesia; POD1*, *postoperative day 1*.

The difference in average pain intensity between the groups is not constant over time (p-value for the interaction term time by group equals 0.0343). Table 3 presents for each time point, the average difference between the groups (standard error), and a Bonferonni corrected p-value. Fig 4 illustrates the pain intensity rated by the NRS scale at all time points. At discharge, patients receiving an ultrasound-guided forearm nerve block report significantly lower pain intensities as compared to patients receiving a forearm IVRA (on average 1.76 points lower, for multiple testing corrected CI (-2.92,-0.59), p<0.001). The difference in pain intensity at the start of the surgery, on average -0.98, becomes not statistically when correcting for multiple testing (p = 0.0345 uncorrected, p = 0.1379 with Bonferonni correction). The pain intensities during the surgery and at POD1 are not statistically significant. During the surgery, the difference between the two groups equals -2.04 (for multiple testing corrected CI (-5.43, 1.36)), but because of the low number of patients with an NRS score during surgery, this difference is not statistically significant (p = 0.5281).

**Table 3:** Estimate (standard error) of the average difference in pain intensity between the ultrasound-guided forearm nerve block and the forearm IVRA group, obtained from a linear mixed model including fixed effects for the group, time their interaction, and a random patient effect and excluding pain at block procedure.

	Forearm IVRA	Peripheral nerve block	Mean Differences (&S.E.)	p-value (Bonferonni correction)
Block procedure	2.31 ± 2.93	2.10 ± 2.77		
Start surgery	2.70 ± 2.89	1.72 ± 2.71	-0.98 (0.46)	0.1379
During surgery (after 10 minutes)	2.25 ± 2.63	0.44 ± 0.72	-2.04 (1.34)	0.5281
At discharge	2.38 ± 2.23	0.62 ± 1.16	-1.76 (0.46)	<0.001
Postoperative day 1	2.80 ± 2.54	2.74 ± 2.19	-0.06 (0.46)	1.0000

## Discussion

In this prospective, randomized, observed-blinded superiority trial, peripheral nerve block, i.e. the combination of an ultrasound-guided median and ulnar nerve block at mid-forearm level supplemented with circumferential, subcutaneous infiltration of the radial side of the wrist, was superior compared to forearm IVRA in providing a surgical block in patients undergoing carpal tunnel release. In total, 7 (14%) of the peripheral nerve blocks were evaluated as incomplete, compared to 17 (34%) of the forearm IVRA. None of the participants required conversion to general anaesthesia.

Patient satisfaction with perioperative analgesia and surgeon satisfaction with the visibility in the surgical field was slightly higher after peripheral nerve block.

Our results are in line with literature on ultrasound-guided peripheral nerve blocks in the forearm. In a retrospective study, Mariano et *al*. [4] also reported no intraoperative conversions to general anaesthesia or postoperative complications after ultrasound-guided median and ulnar nerve block at the mid-forearm level for carpal tunnel release. Ince et *al*. [15] demonstrated a success ratio of 100% after combined ultrasound-guided peripheral median and radial nerve block (superficial, sensory branch) for hand surgery, although they evaluated the success of the block using a cold sensation score. Soberon et *al*. [7] reported one conversion to general anaesthesia out of 30 patients undergoing combined ultrasound-guided peripheral median, ulnar, and radial nerve block at the mid to proximal forearm region and concluded that distal peripheral nerve blocks could serve as alternatives to proximal brachial plexus block in patients undergoing hand surgery. In a retrospective study conducted at the same institution, Soberon et *al*. [7] found a success rate of 90% after peripheral nerve block as a primary anaesthetic for hand and wrist surgery.

Despite the superiority of forearm nerve block over forearm IVRA in providing a surgical block, still, 14% of all patients required additional intravenous opioids. There are several explanations for the lower than predicted success rate of both blocks. First, the definition of a successful block varies in the literature. For example, Chiao et al. [16] reported a success rate of 100% for forearm IVRA (i.e. no need for conversion to general anaesthesia) but also the requirement of a strong intravenous opioid in 29% of all patients with a forearm IVRA. According to our definition, the block success rate would be only 71% in the latter study. Second, also differences in type and dose of local anaesthetic used to perform the blocks may have influenced the success rate of these blocks. For example, Soberon et al. [7] reported a success rate of 96% of peripheral nerve block in patients undergoing ‘hand surgery’. In this trial, peripheral nerve blocks were performed with 5 to 10 mL of 0.5% bupivacaine per nerve (up to a total of 30 mL). Finally, also the type of surgical procedure may have influenced the block success rate. In a retrospective analysis, Soberon et al. [17] concluded that 2 of the 3 forearm block “failures” in that investigation occurred in patients whose surgical incision extended into the wrist and forearm.

A potential disadvantage of the forearm nerve block compared to IVRA or proximal brachial plexus block is the necessity of more than one needle puncture. However, procedural pain or discomfort elicited by the performance of the block was low in both treatment groups and not significantly different.

Total OR time was similar in both study groups (forearm IVRA: 25 min vs forearm nerve block: 26 min, p = 0.95). Thus, despite the use of a block room to perform all forearm nerve blocks preoperatively, operating room efficiency wasn’t improved in the peripheral nerve group. In contrast, Mariano et al. [4] have demonstrated that ultrasound-guided nerve blocks performed preoperatively reduce anaesthesia-controlled time significantly compared to IVRA. This apparent discrepancy could be attributed to our efforts to blind all observers. The surgeon was asked to leave the OR between two surgeries and a single-cuff tourniquet on the forearm was placed in all patients, likely resulting in a delay primarily in the peripheral nerve group. All patients in the forearm IVRA group were also already prepared in the regional anaesthesia block room with intravenous access in both arms. This more than likely resulted in a shorter OR time in the forearm IVRA study group compared to a normal forearm IVRA procedure). Finally, forearm IVRA can improve OR efficiency compared to conventional IVRA since forearm IVRA has no minimal tourniquet inflation time [11–13].

Despite a triple block, including an ultrasound-guided ulnar and median nerve block and additionally circumferential subcutaneous infiltration at the radial side of the wrist to block terminal sensory branches of the radial, lateral antebrachial cutaneous nerve and medial antebrachial cutaneous nerves that may innervate a part of the incision area, still, 14% of blocks were evaluated as inadequate. This observation may be explained by the insufficient time interval between block placement and start surgery in some cases. Another explanation is the inability to block all terminal sensory branches of other cutaneous branches than the ulnar and median nerve that may innervate the incision area due to large interindividual anatomical variability. Regardless, all of these patients with an incomplete block could be treated successfully with additive intravenous analgesics without the need for conversion to general anaesthesia.

Special attention was given to treatment homogeneity in this study by including only patients undergoing unilateral carpal tunnel release and by treating all patients with a combination of intravenous paracetamol, ketorolac, and dexamethasone. Ketorolac was added for its anti-inflammatory properties and dexamethasone for its positive effect on the extension of the duration of locoregional anaesthesia and the prevention of postoperative nausea and vomiting [18].

The present study also contains some limitations. First, because of the use of ultrasound in only one group and the different number of injections, patients could not be blinded to group allocation (observer-blinded study). Second, the time needed to place the block, and the onset time of the sensory blocks was not evaluated here. These parameters, however, can be relevant to measure efficiency in the OR associated with the cost-effectiveness of both techniques. Third, a significant part of all carpal tunnel releases is performed under only local anaesthesia. Ideally, the quality of this type of block, the satisfaction of patients with the quality of this type of block, and the cost-effectiveness of this technique should also have been compared with IVRA and peripheral nerve block in a three-arm study. Fourth, the reported significance levels for the secondary endpoints considered in the study are not corrected for multiplicity. Consequently, the risk of a false positive conclusion is increased. Finally, the forearm IVRA technique is still not widely adopted. However, because of the higher safety profile of this technique and our experience with this technique, we chose to compare peripheral nerve block with forearm IVRA.

In conclusion, our results suggest that ultrasound-guided forearm nerve block is superior compared to forearm IVRA in providing a surgical block and in postoperative pain relief at discharge in patients undergoing carpal tunnel release. Future studies are necessary to evaluate the cost-effectiveness of both techniques and to compare those techniques with local anaesthesia for minor hand- and wrist surgery.

## Supporting information

S1 Checklist CONSORT 2010 checklist of information to include when reporting a randomised trial. (DOC)

S1 Table Maximum likelihood estimates (standard errors) for the parameters in the lineair mixed model, including fixed effects for group, time, their interaction and a random patient effect. For each fixed effect the p value for the overall hypothesis test is reported (F-test).
(DOCX)

S1 File Study protocol. (DOCX)

S2 File Protocol English translation. (DOCX)
