# Supplementary material for: Efficacy of ultrasound-guided forearm nerve block versus forearm intravenous regional anaesthesia in patients undergoing carpal tunnel release: A randomized controlled trial
Source: PLoS One. 2021 Feb 19;16(2):e0246863. doi: 10.1371/journal.pone.0246863 (PMC7895351; doi:10.1371/journal.pone.0246863)
Supplement: S1 Table — For each fixed effect the p value for the overall hypothesis test is reported (F-test). (DOCX) [file pone.0246863.s002.docx]

S1 Table : Maximum likelihood estimates (standard errors) for the parameters in the lineair mixed model, including fixed effects for group, time, their interaction and a random patient effect. For each fixed effect the p value for the overall hypothesis test test is reported (F-test).

| **Fixed Effects** estimate (s.e.) |  | **Random effect** estimate |
| --- | --- | --- |
| Intercept 2.74 (0.33)  *Time*  Start -1.02 (0.42)  During surgery -2.20 (0.80)  At discharge -2.12 (0.42)  POD1 /  *Group*  IVRA 0.06 (0.46)  Ultrasound /  *Group x Time*  Start surgery  IVRA 0.92 (0.60)  Ultrasound /  During surgery  IVRA 1.97 (1.40)  Ultrasound /  At discharge  IVRA 1.70 (0.59)  Ultrasound /  POD1  IVRA /  Ultrasound / |  | Variance random intercepts 0.8583  Residual error variance 4.4418 |
|  |  |  |
|  |  | **Test of fixed effects** p value  Time 0.0004  Group 0.0047  Group x time 0.0343 |
