# Supplementary material for: Efficacy of ultrasound-guided forearm nerve block versus forearm intravenous regional anaesthesia in patients undergoing carpal tunnel release: A randomized controlled trial
Source: PLoS One. 2021 Feb 19;16(2):e0246863. doi: 10.1371/journal.pone.0246863 (PMC7895351; doi:10.1371/journal.pone.0246863)

**Protocol:**

**Efficacy of ultrasound-guided peripheral nerve block versus forearm Bierse block in patients undergoing carpal tunnel release: A randomized controlled trial.**

1. **Introductie en rationale**

Introductie:

Handchirurgie gebeurt in België onder verscheidene vormen van anesthesie (algemene anesthesie, intraveneuze regionale anesthesie en locoregionale anesthesie). Locoregionale anesthesie en intraveneuze regionale anesthesie zijn met name populair gezien patiënten postoperatief vaak snel ontslagklaar zijn na deze types van anesthesie.

Uit de literatuur blijkt dat een onderarm IVRA of mini-bierse block een effectieve en veilige vorm van anesthesie is voor kleine handchirurgie. Doordat bij een mini-bierse blok de dosis van het lokaal anestheticum kan gehalveerd worden ten opzichte van het klassieke bovenarm bierse blok, wordt het risico op lokaal anestheticum toxiciteit voor de patiënt geminimaliseerd. De procedure van het mini-bierse blok wordt uitvoerig beschreven in de 7^e^ alinea van dit protocol.

Een distaal perifeer zenuw blok waarbij onder echogeleiding de nervus medianus en de nervus ulnaris worden verdoofd is een andere vaak toegepaste vorm van locoregionale anesthesie voor kleine handchirurgie. Deze procedure wordt uitvoerig beschreven in de 7^e^ alinea van dit protocol. Uit onderzoek blijkt dat een distaal perifeer zenuw block geassocieerd met kortere chirurgische operatietijd in vergelijking met een IVRA ^1^.

Een carpaal tunnel release is een frequent uitgevoerde operatie in het JESSA ziekenhuis. In het JESSA-ziekenhuis wordt deze standaard uitgevoerd met een mini-bierse block.

Tot op heden is de analeptische effectiviteit van het mini-bierse blok nog niet vergeleken met het distaal perifeer zenuwblok. Het primaire doel van deze studie zal dan ook zijn:

Het bestuderen en vergelijken van de analgetische effectiviteit van het mini-bierse blok ten opzichte van het distaal perifeer zenuwblok in een groep van patiënten die een carpaal tunnel release ondergaan. Onze hypothese is dat het distaal perifeer zenuwblok superieur is op vlak van analgetische effectiviteit ten opzichte van het mini-bierse blok.

Secundaire onderzoeksdoelen zijn het bestuderen van de patiënttevredenheid met de anesthesie en tevredenheid van chirurg met de chirurgische condities, de pijnscore bij incisie en per 10 minuten operatieduur (zowel operatiewonde als tourniquet), de postoperatieve pijnscore bij ontslag en op dag 1 na de operatie, het ongemak van het plaatsen van het blok en de chirurgische tijd/ tijd in operatiezaal.

Hypothese:

- Een distaal perifeer onderarm zenuw block geeft superieure pijnstilling ten opzichte van een ‘mini-Bier block’.

1. **Onderzoekers**

Dr. Kristof Nijs, Dr. Hassanin Jalil, Prof. Dr. Björn Stessel

Dienst anesthesie, Jessa ziekenhuis, Hasselt, België

Dr. Gerrit De Wachter

Dienst orthopedie, Jessa ziekenhuis, Hasselt, België

1. **Sponsor**

Deze klinische studie ontvangt geen ondersteuning van een medische firma betreffende financiering, medicatie of materialen.

1. **Eindpunten**

Resultaten zullen beoordeeld worden op dag 0 tijdens de opname in het ziekenhuis en telefonisch op dag 1. Resultaten op het domein van pijn en tevredenheid van patiënt en chirurg zullen beoordeeld worden.

Primaire uitkomstmaat:

1. Kwaliteit van het blok. dit wordt uitgedrukt als compleet of incompleet:
   1. Compleet is gedefinieerd als
      1. graad 1 (volledige motor en sensorische blokkade (m.b.v. pincet) in het medianus en ulnaris gebied)
      2. graad 2 (partiële motor blokkade, geen pijn of diepe druk gevoeligheid)
   2. Incompleet wordt gedefinieerd als
      1. graad 3 (milde pijn wegens operatie met noodzaak tot rescue lokale of opioïde analgesie, partiële motor blokkade,)
      2. graad 4 (onvolledige motor en sensorische blokkade met noodzaak tot sedatie/conversie naar algemene anesthesie).

De primaire uitkomstmaat zal beoordeeld worden door de chirurg (Dr. De Wachter)op het moment dat de patiënt ontsmet is en volledig chirurgisch is afgedekt. Het betreft een observer-blinded studie. In praktijk zal de beoordeling gebeuren 5 minuten na het toedienen van het lokaal anestheticum bij een mini-bierse block of een 30-tal minuten na toedienen van lokaal anestheticum bij een perifeer zenuw blok.

Secundaire uitkomstmaten:

Volgende uitkomsten gaan ook bekeken worden

- Pijnscore bij injectie/plaatsing anesthesie techniek gemeten met een 11 punten ‘Numeric Rating Scale’ (NRS: waar 0 = geen pijn en 10 = ergste pijn mogelijk).
- Pijnscore bij incisie (start operatie) gemeten met een 11 punten ‘Numeric Rating Scale’ (NRS: waar 0 = geen pijn en 10 = ergste pijn mogelijk).
- Intra-operatieve tourniquet pijnscore (elke 10 min NRS score, pijn uitgelokt door de tourniquet)
- Gemiddelde postoperatieve pijnscore gemeten met een 11 punten ‘Numeric Rating Scale’ (NRS: waar 0 = geen pijn en 10 = ergste pijn mogelijk) bij ontslag en op dag 1 (telefonische follow-up).
- Gebruik van postoperatieve pijn medicatie (rescue medicatie) (Paracetamol en/of ibuprofen) op dag 0 en 1 postoperatief.
- Tijd in operating theatre (startend bij binnenkomst operatiezaal tot verlaten van de operatiezaal).
- Chirurgische tijd (incisie tot laatste steek)
- Tevredenheid van chirurg gebruikmakend van een “seven-point Likert scale” (op een schaal van 1 op 7 zijnde absoluut niet tevreden, 4 op 7 zijnde neutrale mening en 7 op 7 zijnde zeer tevreden) over de chirurgische condities. Deze score wordt postoperatief afgenomen.
- Tevredenheid van de patiënt gebruikmakend van een “seven-point Likert scale” (op een schaal van 1 op 7 zijnde absoluut niet tevreden, 4 op 7 zijnde neutrale mening en 7 op 7 zijnde zeer tevreden) op dag 1 postoperatief.

1. **Opzet van de studie**

Het betreft een onderzoeker-geïnitieerd, monocentrisch, observer-blinded, prospectief, gerandomiseerd onderzoek, waarbij 2 behandelgroepen worden vergeleken. Het betreft een superiority trial bij patiënten die carpaal tunnel release in dag behandeling ondergaan.

Groep 1: mini-Bierse Block (standaard therapie in het JESSA ziekenhuis)

Groep 2: distaal perifeer zenuwblock onder echogeleiding

Deze studie wordt uitgevoerd volgens de verklaring van Helsinki en zal door de ethische commissie

van het Jessa ziekenhuis goedgekeurd worden vooraleer ze van start kan gaan. Schriftelijk informed

consent’ wordt van alle deelnemers afgenomen bij aanvang van de studie.

**Steekproeftrekking:**

Randomisatie vindt plaats middels een van tevoren door de computer gegenereerde lijst (zie onder).

**Tijdschema:**

Het onderzoek duurt ongeveer 5 maanden, afhankelijk van het aanbod van deze patiënten kan deze termijn langer duren (maximaal 10 maanden).

1. **Studie populatie**

## 6.1 Populatie

De studie betreft patiënten die op het dagcentrum ambulant geopereerd worden voor een carpaal tunnel release. Er is gekozen voor deze pathologie:

- om enerzijds de 2 studiegroepen zo homogeen mogelijk te maken
- omdat een carpaal tunnel release veelvuldig wordt uitgevoerd in het JESSA ziekenhuis

## 6.2 Inclusie criteria:

- - ≥18 jaar
  - ASA 1 -3
  - Patiënt gepland voor electief carpaal tunnel release onder regionale anesthesie (Distaal perifeer zenuw block of IVRA).

## 6.3 Exclusie criteria:

- - Bilaterale chirurgie
  - BMI ≥ 40 kg/m²
  - Infectie in het gebied voor de perifere zenuw block injecties.
  - Bestaande neurologische aandoeningen
  - Chronische pijn symptomen
  - Opioïd gebruik in de afgelopen 3 maanden
  - Diabetes mellitus type 1 (insuline dependente diabetes mellitus)
  - Diabetes mellitus met orgaan schade tot gevolg (vergevorderde diabetes mellitus)
  - Allergie aan lokale anesthetica
  - Stollingsstoornissen
  - Chirurgie in de voorgeschiedenis aan dezelfde arm.
  - Onvermogen tot begrijpen van het studie-opzet

## 6.4 Sample Size Calculation

Sample size was determined for the primary study outcome with the aim to reject the superiority of the distal peripheral nerve block compared to forearm intravenous regional anesthesia. Based on a retrospective analysis of unpublished data from our hospital, we assume that 75% of patients will have a complete block (grade 1 or2) after a forearm IVRA. Based on a previous study, we assume that 95% of patients will have a complete block (grade 1 or2) after a distal peripheral nerve block (4). By using a binary outcome, we determined the sample size for each group to be 47 (α=0.05, power=0.80). To account for a possible 6% drop-out rate, the sample size was increased to 50 patients per group.

1. **Studie procedure**

**Pre-operatieve fase**

De informatiebrief en het informed consent formulier wordt reeds tijdens de preoperatieve consultatie door de chirurg meegegeven aan de patiënt. Bij aankomst op de dagkliniek wordt elke patiënt die het informed consent formulier heeft ondertekend, geïncludeerd in de studie, nadat is nagegaan of er geen exclusiecriteria zijn. Op basis van de randomisatielijst wordt de patiënt ingedeeld in 1 van de 2 studiegroepen. De NRS-score en het gebruik van het case report form (CRF) worden in detail uitgelegd door een lid van het onderzoeksteam, waarna de patiënt de baselinegegevens invult op het CRF. Dit bestaat uit de demografische gegevens (leeftijd, gewicht, lengte) en de pre-operatieve NRS-score.

Alle zenuwblokkades worden uitgevoerd door slechts 4 anesthesisten, bedreven in het plaatsen van zenuwblokkades, om grote interindividuele verschillen op het vlak van anesthesie te voorkomen. Voor aanvang van anesthesie, ontvangen alle deelnemende patiënten een intraveneuze toegang, supplementaire zuurstoftoediening en standaard monitoring (niet-invasieve bloeddrukmeting, elektrocardiogram en saturatiemeting). Sedatie wordt standaard preoperatief en peroperatief niet gegeven tijdens dag chirurgie.

De chirurg en de studiemedewerkers die de uitkomstmaten bij de patiënt bevragen zullen niet op de hoogte zijn van het type chirurgie die de patiënt heeft ondergaan (zal dus ook niet aanwezig zijn tijdens het plaatsen van het blok).

Preoperatief wordt een intraveneus infuus met NaCl 0,9% gestart in de contralaterale arm. Zuurstofsaturatie en hartritme worden gevolgd via pulse oxymetrie. Op elk ogenblik tijdens de procedure wordt verbaal contact met de patiënt onderhouden.

1. ***Studie-arm: distaal perifeer zenuwblok***

Het distaal perifeer zenuwlok zal preoperatief uitgevoerd worden in de lokale prikzaal, gelegen in de nabijheid van de ontwaakruimte een 30-tal minuten voor de operatie.

Het distaal perifeer zenuwblock bestaat uit het verdoven van de n. medianus en de n. ulnaris (de n. radialis zit niet in het operatiegebied). De medicatie die nabij de zenuwen geïnjecteerd wordt is 10ml linisol 2% om alzo een snelle en kortdurende werking te verkrijgen. Hierbij wordt 3ml linisol 2% rond de n. medianus verdeeld, 3ml linisol 2% rond de n. ulnaris en 4ml linisol 2% rond de perifere distale zenuwtakken. Voor het zenuwblock wordt gebruik gemaakt van een General Electric LOGIQe toestel en een 12 MHz lineaire echotransducer met een voetafdruk van 4 cm. De echoprobe wordt afgekleefd met steriele Tegaderm® folie. Er wordt gebruik gemaakt van steriele echogel.

1. ***Controle-arm: mini-Bierse blok***

Het mini-Bierse blok zal preoperatief uitgevoerd worden in de operatiezaal, een 5-tal minuten voor start van de operatie.

Een voorarm intraveneuze regionale anesthesie (IVRA) ofwel mini-Bierblock bestaat uit het aanleggen van een intraveneuze toegangsweg (met slotje, geen infuus) op de handrug van de te opereren hand. Na bekomen van deze toegangsweg wordt een knelband t.h.v. de voorarm aangelegd en uitgewindeld, vooraleer ze opgelaten wordt. Dit heeft een ”bloedvrije” voorarm tot resultaat, waarna intraveneus via dit slotje 25ml linisol 0.5% wordt geïnjecteerd. De knelband blijft 10 minuten aan om postoperatief, na lossen van de knelband, systemische toxiciteit van het lokaal anestheticum te voorkomen.

**Per-operatieve fase:**

Nadat de patiënt volledig geïnstalleerd, ontsmet en afgedekt is, komen chirurg en geblindeerde studiemedewerker de operatiezaal binnen. De kwaliteit van het blok zal door de chirurg worden bepaald voor start van operatie met behulp van pincetprik in de dermatomen van n. ulnaris en n. medianus gebied (zie boven). Indien voldoende blok (graad 1 of 2) zal de operatie starten. Bij onvoldoende blok (graad 3 of 4) zal het blok verdiept worden: initieel toediening lokaal anestheticum ter hoogte van operatiegebied of intraveneuze opioiden (alfentanil of sufentanil) (Graad III). Indien ook dit onvoldoende is, wordt sedatie/ conversie naar algehele anesthesie toegepast (Graad IV).

Peroperatief wordt paracetamol 15mg/kg (max 1gram) en taradyl 0.5mg/kg (max 30mg) gegeven (indien geen contra-indicaties aanwezig zijn). Omwille van het positieve effect van dexamethasone op de verlenging van een locoregionale block en zijn positief effect op het voorkomen van postoperatieve nausea en braken (PONV) krijgen alle patiënten (zowel distaal perifeer zenuw block en IVRA) dexametasone 0.1mg/kg (max 5mg) toegediend (indien er geen contra-indicaties aanwezig zijn).

Alle secundaire uitkomstmaten zullen door de studiemedewerker geregistreerd worden via bevraging van patiënt en chirurg en via bijhouden van tijden in operatiezaal/chirurgische tijd.

**Postoperatieve fase:**

Postoperatief in het ziekenhuis wordt paracetamol 15mg/kg (max 1gr) 1x/6u en taradyl 0.5mg/kg (max 30mg) 1x/8u voorgeschreven met contramal als rescue medicatie.

Bij ontslag zal de NRS pijnscore worden afgenomen bij de patiënt.

Postoperatief thuis wordt paracetamol 15mg/kg (max 1gr) 1x/6u zo nodig en ibuprofen 600mg 1x/8u zo nodig voorgeschreven voor de pijn.

Op dag 1 postoperatief wordt de patiënt telefonisch gecontacteerd en bevraagd naar tevredenheid met anesthesie, gebruik van pijnmedicatie en NRS pijn score.

Alle data verzameling gebeurt door éénzelfde persoon die geblindeerd is voor de groep allocatie.

1. **Randomisatie en blindering**

Participants will be randomly assigned to 1 of 2 study groups of 50 subjects each: ultrasound-guided peripheral nerve block group or forearm IVRA group. Randomization will be performed using a computer-generated random allocation sequence, created by the study statistician. Allocation numbers will be sealed in opaque envelopes, which will be opened in sequence by an independent anaesthesiologist who is not involved in the assessment of outcomes. Outcome-assessors (surgeon and study assistant) will be blinded to treatment allocation (observer-blinded study).

Het bekomen van blindering is zeer moeilijk gezien de zeer opvallende verschillen tussen beide technieken. Echter, een preoperatief geplaatste additionele intraveneuze toegangsweg (met slotje, geen infuus) in de hand van de operatiezijde zal zorgen voor betere blindering bij de outcome-assessors. Ook wordt op de normale plaatsen waar een perifeer zenuwblock geplaatst wordt, preoperatief bij alle patiënten ontsmet met kleurstof om outcome-assessors te blinderen. Bij aankomst van patiënt in operatiezaal wordt de chirurg 5 minuten gevraagd om even de operatiezaal te verlaten om al dan niet geblindeerd de IVRA medicatie te kunnen inspuiten in de operatiezaal. Nadat de patiënt volledig geïnstalleerd, ontsmet en afgedekt is, komen chirurg en geblindeerde studiemedewerker de operatiezaal pas binnen.

**Veiligheidsrapportage**

Alle mogelijke complicaties worden preoperatief uitgelegd aan de deelnemende patiënten. Er moet benadrukt worden dat beide interventies, zowel een distaal perifeer zenuwblock en een intraveneuze regionale anesthesie (IVRA) techniek gangbare technieken zijn om de anesthesie te bekomen bij electieve handchirurgie in dagziekenhuis setting.

1. **Statische analyse**

Beschrijvende statistiek zal gepresenteerd worden als frequenties en percentage van het totaal aantal mensen voor categorische variabelen, terwijl numerieke variabelen gepresenteerd zullen worden als gemiddelde ± SD. Groepsvergelijkingen worden uitgevoerd met behulp van een Chi-square test (of wanneer nodig Fisher’s Exact test) voor frequenties. Afhankelijk van normaliteit zal een Mann-Witney U test gebruikt worden voor niet-normaal verdeelde numerieke variabelen of een Student’s t-test voor normaal verdeelde numerieke variabelen. Een Mixed-model analyse zal gebruikt om te corrigeren voor de herhaalde metingen van de NRS pijnscore.

Een gemiddeld verschil van 1,3 punten of meer op de NRS pijnscore beschouwd als een klinisch relevant resultaat.

Een p-waarde < 0.05 wordt beschouwd als statistisch significant, terwijl p < 0.10 beschouwd wordt als een tendens.

1. **Ethische overwegingen**

Beide interventies, zowel een distaal perifeer zenuwblock en een intraveneuze regionale anesthesie (IVRA) techniek, zijn gangbare technieken om anesthesie te bekomen bij electieve handchirurgie in dagziekenhuis setting. We willen met dit studie-opzet enkel kijken welke techniek de beste van de twee is.

**Referenties:**

1. Mariano ER, Lehr MK, Loland VJ, Bishop ML. Choice of loco-regional anesthetic technique affects operating room efficiency for carpal tunnel release. J Anesth. 2013 Aug;27(4):611–4.

2. Wong J, Tong D, De Silva Y, Abrishami A, Chung F. Development of the functional recovery index for ambulatory surgery and anesthesia. Anesthesiology. 2009 Mar;110(3):596–602.

3. van Agt HM, Essink-Bot ML, Krabbe PF, Bonsel GJ. Test-retest reliability of health state valuations collected with the EuroQol questionnaire. Soc Sci Med 1982. 1994 Dec;39(11):1537–44.

4. Soberón J., Crookshank J., Nossama B., Elliott C., Sisco-Wise L., Duncan S., Distal peripheral nerve blocks in the forearm as an alternative to proximal brachial plexus blockade in patients undergoing hand surgery: a prospective and randomized pilot study. J Hand Surg Am. 2016 Oct; 41(10):969-977

Bijlages:

1. Numeric Rating Scale


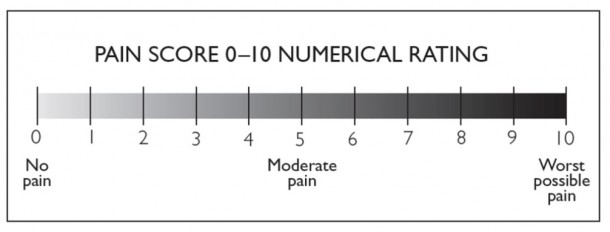


1. Seven-point Likert scale:


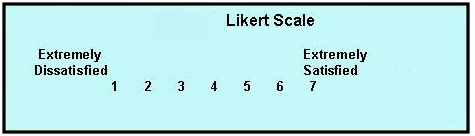

Supplement: S1 File — (DOCX) [file pone.0246863.s003.docx]
