# Supplementary material for: Efficacy of ultrasound-guided forearm nerve block versus forearm intravenous regional anaesthesia in patients undergoing carpal tunnel release: A randomized controlled trial
Source: PLoS One. 2021 Feb 19;16(2):e0246863. doi: 10.1371/journal.pone.0246863 (PMC7895351; doi:10.1371/journal.pone.0246863)

**Protocol:**

**Efficacy of ultrasound-guided peripheral nerve block versus forearm Bierse block in patients undergoing carpal tunnel release: A randomized controlled trial.**

1. **Introduction and hypothesis**

Introduction:

In Belgium, several ways of anesthesia are used to perform hand surgery, being general anesthesia, intravenous regional anesthesia as well as locoregional anesthesia. Locoregional anesthesia and intravenous regional anesthesia are often performed since patients can be discharged from the hospital more rapidly.

A forearm intravenous regional anesthesia (IVRA; mini-Bier’s block) is an effective and safe technique to perform hand surgery, especially since the dose of the used anesthetic is reduced compared to a regular upper arm Bier’s block, leading to a reduced risk of local anesthetic toxicity.

An ultrasound-guided distal peripheral nerve block is often used for hand surgery, in which the nervus medianus and the nervus ulnaris can be affected locally. Research showed that this technique is associated with a reduced surgical procedure time when compared to intravenous regional anesthesia (1).

In the Jessa Hospital, a carpal tunnel release is often performed using the forearm intravenous regional anesthesia. However, to date, the analeptic effectivity of the forearm intravenous regional anesthesia has not been compared to the distal peripheral nerve block.

The primary aim of this study is to compare the analgesic effectivity of the forearm intravenous regional anesthesia versus the ultrasound-guided distal peripheral nerve block. Our hypothesis is that the latter technique is superior. A blinded surgeon will determine the analgesic effectivity of these techniques (graded as “complete” or “incomplete”) and divide the effectivity in 4 grades. Grade 1 and grade 2 are considered “complete” blockade, while grade 3 and grade 4 are considered “incomplete” blockade. Grade 1: complete motor and sensory blockade, grade 2: partial motor blockade but no pain or deep pressure sensitivity, grade 3: partial motor blockade with mild pain requiring rescue local or opioid analgesia, grade 4: incomplete motor- and sensory blockade requiring sedation/conversion to general anesthesia).

Secondary study outcomes are patient’s pre-, intra-, and post-operative pain scores at several time points, measured via an 11-point Numeric Rating Scale (NRS) in which number 0 means “no pain at all”, number 5 means “moderate pain” and number 10 means “worst possible pain”.

General patient satisfaction as well as satisfaction of the surgeon regarding the used technique will be measured via a 7-point Likert Scale, in which number 1 means “extremely dissatisfied” and number 7 means “extremely satisfied”.

Furthermore, the time spend in the operating room, the time of surgical procedure and the use of post-operative medication use will be recorded as well.

1. **Researchers**

Dr. Kristof Nijs, Dr. Hassanin Jalil, Prof. Dr. Björn Stessel

Anesthesiology, Jessa hospital, Hasselt, België

Dr. Michael Vaninbroukx

Orthopedic Surgery, Jessa ziekenhuis, Hasselt, Belgium

1. **Funding**

This study received no funding.

1. **Outcome measures**

Results will be evaluated on day 0 during admission to hospital and on day 1 with a telephone call.

Primary outcome:

The primary outcome of this study is to compare the analgesic effectivity of the forearm intravenous regional anesthesia versus the ultrasound-guided distal peripheral nerve block. A blinded surgeon will determine the analgesic effectivity of these techniques after the patient is fully ready and surgically covered as this is an observed blinded study. The surgeon will grade the anaesthesia as “complete” or “incomplete” and divide the effectivity in 4 grades. Grade 1 and grade 2 are considered “complete” blockade, while grade 3 and grade 4 are considered “incomplete” blockade. Grade 1: complete motor and sensory blockade with a pincet in the medianus and ulnaris area, grade 2: partial motor blockade but no pain or deep pressure sensitivity, grade 3: partial motor blockade with mild pain requiring rescue local or opioid analgesia, grade 4: incomplete motor- and sensory blockade requiring sedation/conversion to general anesthesia).

Secondary outcomes:

- Pre-operative: pain score at start of anesthesia technique (measured via an 11-point Numeric Rating Scale)
- Pre-operative: pain score at surgical incision (start of surgical procedure) (11-point Numeric Rating Scale)
- Intraoperative: pain score (11-point Numeric Rating Scale) at every 10 min during the surgical procedure (both surgical pain and tourniquet pain)
- Time the patient is in operating room (start by entering the operating room until leaving the operating room)
- Time of surgical procedure (start at surgical incision until closing of the surgical wound)
- Post-operative: average pain score at hospital discharge and at day 1 after surgery
- Post-operative: use of pain medication (paracetamol and/or ibuprofen) after surgery and at day 1 after surgery
- General satisfaction of the patient with the anesthesia technique (measured via a 7-point Likert scale with 1 absolutely not satisfied, 4 neutral and 7 extremely satisfied)
- Satisfaction of the surgeon with the surgical conditions (7-point Likert scale)

1. **Study design**

In this monocentric, investigator-initiated, observer-blinded, prospective, randomized controlled trial, 2 groups of patients that will undergo carpal tunnel release surgery will be compared.

Group 1: mini-Biers Block (standard of care in JESSA hospital)

Group 2: distal peripheral ultrasound-guided nerve block

This study will be performed according to the Declaration of Helsinki and will be approved by the Ethical Committee of the Jessa Hospital before the start of the study. A written informed consent will be obtained before participation in the study.

Randomization will be performed using a computer-generated random allocation sequence, created by the study statistician. Allocation numbers will be sealed in opaque envelopes, which will be opened in sequence by an independent anaesthesiologist who is not involved in the assessment of outcomes.

1. **Study population**

## 6.1 Population

All patients scheduled for a carpal tunnel release in the jessa hospital are eligible to partipate.

## 6.2 Inclusion criteria:

- - Males and females ≥ 18 years of age
  - American Society of Anesthesiology (ASA) physical status classification: 1-3
  - Patients planned to undergo carpal tunnel release via regional anesthesia (intravenous regional anesthesia or ultra-sound guided peripheral nerve block)

## 6.3 Exclusie criteria:

- - Bilateral surgery
  - BMI ≥ 40 kg/m2
  - Infection in the area of the peripheral nerve block injection site
  - History of neurological conditions
  - Chronic pain symptoms
  - Concomitant use of Opioids in the last 3 months
  - Diabetes mellitus type-1 (insulin dependent diabetes mellitus)
  - Diabetes mellitus with organ damage
  - Oversensitivity to local anesthesia
  - Blood clotting disorder
  - History of any surgical procedure in the arm that needs to be operated
  - Inability to understand and adhere to the study design

## 6.4 Sample Size Calculation

Sample size was determined for the primary study outcome with the aim to reject the superiority of the distal peripheral nerve block compared to forearm intravenous regional anesthesia. Based on a retrospective analysis of unpublished data from our hospital, we assume that 75% of patients will have a complete block (grade 1 or2) after a forearm IVRA. Based on a previous study, we assume that 95% of patients will have a complete block (grade 1 or2) after a distal peripheral nerve block (4). By using a binary outcome, we determined the sample size for each group to be 47 (α=0.05, power=0.80). To account for a possible 6% drop-out rate, the sample size was increased to 50 patients per group.

1. **Study procedures**

**Pre-operatieve fase**

The patient will first receive information about this study during the consultation with the surgeon. After having ample amount of time to consider participation and before the start of the study, written informed consent will be obtained. Afterwards, the patient will be randomized to one of the 2 study groups.

Demographical data such as age, weight and lengthe together with preoperative NRS score will be assessed.

All anesthesia techniques will be performed by 4 anaesthesiologists, to reduce the inter-individual difference in anaesthesia. All patients will receive an intravenous catheter, supplementary oxygen and standard monitoring (non-invasive blood pressure, electrocardiogram and saturation measurements). General sedation is preoperative and perioperative not applied during day surgery.

The surgeon and the researcher will be blinded for the used type of anaesthesia. To reduce a possible deblinding, all patients receive a catheter in the dorsal vein of the hand that will undergo surgery and all patients will receive disinfection on the specific locations on the arm where the ultrasound-guided peripheral nerve block would be performed. In that way, both surgeon and researcher are blinded.

1. ***Distal peripheral nerveblock***

The distal peripheral nerve block will be performed in the local prick area 30 minutes before the surgery. A General Electric LOGIQe device and a 12 MHz lineair echotransducer with a foot of 4 cm will be used to locate the nervus medianus and the nervus ulnaris. In total, 10ml linisol 2% will be used to affect the nerves (3ml linisol 2% around the nervus medianus, 3ml linisol 2% around the nervus ulnaris and 4ml linisol 2% around the peripheral distal nerves).

The echoprobe is treated with sterile Tegaderm® foil and sterile echogel is used.

1. ***Mini-Bier block***

The Mini-Bier block will be performed in the opeation theathre, 5 min prior to the start of the surgery. An intravenous catheter will be placed in the dorsal vein of the hand that will undergo surgery. Also, a tourniquet will be placed on the forearm, after which the anesthetic can be administered via the dorsal vein catether (25ml linisol 0.5%).

**Peri-operative phase:**

Before the start of the surgery, the surgeon will assess the quality of the block (primary outcome) in the nervus ulnaris and nervus medianus, using a forceps. The quality of the block will be graded “complete” (grade 1 and grade 2) or “incomplete” (grade 3 or grade 4). In case of grade 1 or grade 2, the surgical procedure can start. In case of grade 3, the blockade will be enhanced (local anesthetic for peripheral nerve block or intravenous opioid administration (alfentanil or sufentanil) for IVRA). In case of grade 4, sedation/conversion to general anesthesia will be performed.

Per-operative paracetamol administration 15mg/kg (max 1gram) and taradyl 0.5mg/kg (max 30mg) will be supplied (except when there are contraindications). All patients (both distal peripheral nerve block and IVRA) will receive dexamethasone 0.1mg/kg (max 5mg) (except when there are contraindications) due to the positive effect of dexamethasone on the extension of the locoregional anesthesia and the prevention of post-operative nausea and vomiting.

All secondary outcome measures will be investigated by the blinded researcher via questioning the patient’s pain scores (measured via an Numeric Rating Scale) at several time points before, during and after surgery, and the satisfaction of both patient and surgeon (measured via a 7-point Likert Scale).

**Postoperative phase:**

Post-operative pain medication in the hospital includes paracetamol 15mg/kg (max 1gr) 1x/6h and taradyl 0.5mg/kg (max 30mg) 1x/8h, while contramal serves as rescue medication.

Post-operative pain medication at home includes paracetamol 15mg/kg (max 1gr) 1x/6h or ibuprofen 600mg 1x/8h whenever necessary.

On day 1 after surgery, the patient will be contacted via telephone by the researcher who will question the use of post-operative pain medication, as well as NRS pain scores and patient satisfaction.

1. **Randomisatie en blindering**

Participants will be randomly assigned to 1 of 2 study groups of 50 subjects each: ultrasound-guided peripheral nerve block group or forearm IVRA group. Randomization will be performed using a computer-generated random allocation sequence, created by the study statistician. Allocation numbers will be sealed in opaque envelopes, which will be opened in sequence by an independent anaesthesiologist who is not involved in the assessment of outcomes. Outcome-assessors (surgeon and study assistant) will be blinded to treatment allocation (observer-blinded study).

The surgeon and the researcher will be blinded for the used type of anaesthesia. To reduce a possible deblinding, all patients receive a catheter in the dorsal vein of the hand that will undergo surgery and all patients will receive disinfection on the specific locations on the arm where the ultrasound-guided peripheral nerve block would be performed. In that way, both surgeon and researcher are blinded.

Also, the surgeon and the assessor are asked to leave to operation room and only to enter after the patient is fully installed, desinfected and covered.

**Safety**

All possible complications were explained to the participant preoperatieve. Both interventions, the distal peripheral nerve block and the mini biers block are standard techniques used in the Jessa hospital.

1. **Statistical analysis**

Sample size was determined for the primary study outcome with the aim to reject the superiority of the distal peripheral nerve block compared to forearm intravenous regional anesthesia. Based on a retrospective analysis of unpublished data from our hospital, we assume that 75% of patients will have a complete block (grade 1 or2) after a forearm IVRA. Based on a previous study, we assume that 95% of patients will have a complete block (grade 1 or2) after a distal peripheral nerve block (2). By using a binary outcome, we determined the sample size for each group to be 47 (α=0.05, power=0.80). To account for a possible 6% drop-out rate, the sample size was increased to 50 patients per group.

Descriptive statistics will be presented as frequencies and percentages of the total amount of patients for categorical variables, while numerical variables will be presented as mean ± SD. Group comparison will be performed using Chi-square test (or a Fisher’s Exact test when necessary) for frequencies. Depending on normality, a Mann-Witney U test or a Student’s t-test will be used and a Mixed-model analysis will be used to correct for the multiple measurements of the Numerical Rating Scale (NRS) for pain scores. An average difference of 1.3 points or more on the NRS pain score is considered clinical relevant. A p-value <0.05 is considered statistical significant, while p<0.10 is considered a tendency.

**References:**

1. Mariano ER, Lehr MK, Loland VJ, Bishop ML. Choice of loco-regional anesthetic technique affects operating room efficiency for carpal tunnel release. J Anesth. 2013 Aug;27(4):611–4.

2. Wong J, Tong D, De Silva Y, Abrishami A, Chung F. Development of the functional recovery index for ambulatory surgery and anesthesia. Anesthesiology. 2009 Mar;110(3):596–602.

3. van Agt HM, Essink-Bot ML, Krabbe PF, Bonsel GJ. Test-retest reliability of health state valuations collected with the EuroQol questionnaire. Soc Sci Med 1982. 1994 Dec;39(11):1537–44.

4. Soberón J., Crookshank J., Nossama B., Elliott C., Sisco-Wise L., Duncan S., Distal peripheral nerve blocks in the forearm as an alternative to proximal brachial plexus blockade in patients undergoing hand surgery: a prospective and randomized pilot study. J Hand Surg Am. 2016 Oct; 41(10):969-977

Attachment:

1. Numeric Rating Scale


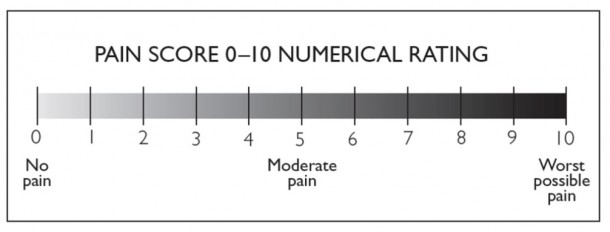


1. Seven-point Likert scale:


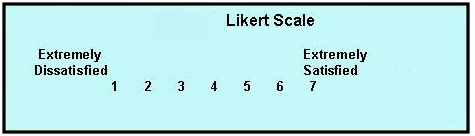

Supplement: S2 File — (DOCX) [file pone.0246863.s004.docx]
